# Prevalence of Mild Cognitive Impairment and Alzheimer’s Disease Identified in Veterans in the United States

**DOI:** 10.3233/JAD-240027

**Published:** 2024-05-28

**Authors:** Byron J. Aguilar, Guneet K. Jasuja, Xuyang Li, Ekaterina Shishova, Natalia Palacios, Dan Berlowitz, Peter Morin, Maureen K. O’Connor, Andrew Nguyen, Joel Reisman, Yue Leng, Raymond Zhang, Amir Abbas Tahami Monfared, Quanwu Zhang, Weiming Xia

**Affiliations:** a Geriatric Research Education and Clinical Center, VA Bedford Healthcare System, Bedford, MA, USA; b The Bedford VA Research Corporation, Inc., Bedford, MA, USA; c Center for Healthcare Organization & Implementation Research, VA Bedford Healthcare System, Bedford, MA, USA; d Section of General Internal Medicine, Boston University Chobanian and Avedisian School of Medicine, Boston, MA, USA; e Department of Health Law, Policy and Management, Boston University School of Public Health, Boston, MA, USA; f Department of Biomedical Engineering, Johns Hopkins University, Baltimore, MD, USA; g Department of Public Health, Zuckerberg College of Health Sciences, University of Massachusetts Lowell, Lowell, MA, USA; h Department of Neurology, Boston University Chobanian & Avedisian School of Medicine, Boston, MA, USA; i Department of Biological Science, Kennedy College of Sciences, University of Massachusetts Lowell, Lowell, MA, USA; j Department of Psychiatry, University of California San Francisco Weill Institute for Neurosciences, San Francisco, CA, USA; k Alzheimer’s Disease and Brain Health, Eisai Inc., Nutley, NJ, USA; l Epidemiology, Biostatistics and Occupational Health, McGill University, Montreal, QC, Canada; m Department of Pharmacology, Physiology and Biophysics, Boston University Chobanian & Avedisian School of Medicine, Boston, MA, USA

**Keywords:** Alzheimer’s disease,, electronic health record, mild cognitive impairment, prevalence, veterans affairs

## Abstract

**Background:**

Diagnostic codes can be instrumental for case identification in Alzheimer’s disease (AD) research; however, this method has known limitations and cannot distinguish between disease stages. Clinical notes may offer more detailed information including AD severity and can complement diagnostic codes for case identification.

**Objective:**

To estimate prevalence of mild cognitive impairment (MCI) and AD using diagnostics codes and clinical notes available in the electronic healthcare record (EHR).

**Methods:**

This was a retrospective study in the Veterans Affairs Healthcare System (VAHS). Health records from Veterans aged 65 years or older were reviewed during Fiscal Years (FY) 2010–2019. Overall, 274,736 and 469,569 Veterans were identified based on a rule-based algorithm as having at least one clinical note for MCI and AD, respectively; 201,211 and 149,779 Veterans had a diagnostic code for MCI and AD, respectively. During FY 2011–2018, likely MCI or AD diagnosis was defined by≥2 qualifiers (i.e., notes and/or codes)≥30 days apart. Veterans with only 1 qualifier were considered as suspected MCI/AD.

**Results:**

Over the 8-year study, 147,106 and 207,225 Veterans had likely MCI and AD, respectively. From 2011 to 2018, yearly MCI prevalence increased from 0.9% to 2.2%; yearly AD prevalence slightly decreased from 2.4% to 2.1%; mild AD changed from 22.9% to 26.8%, moderate AD changed from 26.5% to 29.1%, and severe AD changed from 24.6% to 30.7%

**Conclusions:**

The relative distribution of AD severities was stable over time. Accurate prevalence estimation is critical for healthcare resource allocation and facilitating patients receiving innovative medicines.

## INTRODUCTION

On July 6, 2023, the United States (US) Food and Drug Administration (FDA) granted full approval to the anti-amyloid therapy, lecanemab, for the treatment of Alzheimer’s disease (AD), with recommended initiation in patients with mild cognitive impairment (MCI) or mild dementia stage of AD [1]. AD has been described as a pathophysiological continuum encompassing a preclinical phase with undetectable symptoms, MCI, characterized by very mild symptoms that may not interfere with daily activities, and dementia, which may be classified into mild, moderate, and severe stages that increasingly hinder daily life with disease progression [2, 3]. As therapeutic strategies for AD shift beyond symptom management to slowing of disease progression, diagnosis in the early stages will afford patients and their caregivers more time to plan for care and to identify suitable interventions [4].

The US Department of Veteran’s Affairs projected the prevalence of Veterans with AD receiving care within the Veteran’s Affairs Healthcare System (VAHS) to be 167,954 (95% confidence interval 65,007 to 272,020) in fiscal year (FY) 2022 [5]. Additionally, several studies in the VAHS have relied on diagnostic code-based identification of AD and dementia [6–8]. While use of diagnostic codes in epidemiologic research can be instrumental for case identification, this method is limited by the possibility that codes represent a rule-out examination/diagnostic workup, miscoding/undercoding, and the inability to distinguish between AD stages. Clinical notes may offer more detailed information including disease severity and can serve as a complementary method to diagnostic codes for case identification.

In the current investigation, we aimed to provide estimates of the prevalence of both MCI and AD in the US Veteran population aged 65 years or older by searching clinical notes, in addition to diagnostic codes in the VAHS electronic health record (EHR). We further illustrated the trend of prevalence estimates in the past decade.

## MATERIALS AND METHODS

### Data source and extraction

This was a retrospective analysis of the Veterans Affairs Informatics and Computing Infrastructure (VINCI) database which partners with the Corporate Data Warehouse (CDW) [8]. Health records from Veterans aged 65 years or older were reviewed during fiscal years (FYs) 2010–2019 (October 1, 2009 through September 30, 2019); we have previously described a rule-based method for identification of individuals with MCI (all-cause) or AD using clinical notes from the VAHS EHR [9]. Briefly, Text Integration Utilities (TIU) from the Microsoft Statistical Query Language (SQL) Server relational database system were used to perform key word searches (“Alz*”, “MCI”, “mild cognitive impairment”) of EHR notes. Random subsets of≥100 notes were reviewed manually to determine the positive predictive values (PPVs) of these searches; iterative processes including step-wise exclusions were applied to enhance case identification precision, achieving threshold PPVs of≥80%. Additionally, Veterans with diagnostic codes for MCI (*ICD-9-CM* 331.83; *ICD-10-CM* G31.84) and AD (*ICD-9-CM* 331.0; *ICD-10-CM* G30.X) were identified from EHR structured data. We chose AD diagnostic codes rather than non-specific dementia codes to capture AD-specific clinical workups. This study was approved by the VA Bedford Healthcare System Institutional Review Board and all data were fully de-identified before access.

### Case definitions

The base population consisted of all Veterans who had≥1 VAHS inpatient or outpatient encounter in FYs 2010–2019. Veterans with a date of death (DOD) in the EHR before the end of the FY being analyzed were excluded. A classification algorithm for Veterans recorded with MCI or AD within their notes and/or by codes between FYs 2011–2018 was applied:

Veterans met the case definition for a likely MCI or AD diagnosis if they had≥2 qualifiers (i.e., qualifying notes or codes),≥30 days apart by searching the qualifying clinical encounters in each FY plus 1 FY before and after. The “2 qualifiers” could consist of≥2 qualifying notes from unstructured data,≥2 qualifying codes from structured data, or a combination of≥1 qualifying note plus≥1 qualifying code. This case definition was chosen in order to capture at least two separate clinical encounters by a Veteran during which MCI- or AD-related clinical workups were likely to have taken place.
Veterans did not meet the case definition for MCI or AD if they had only 1 qualifying note or code in the current FY, but were considered to have suspected MCI or AD.

Veterans fulfilling the case definition (2 qualifiers for MCI or 2 qualifiers for AD) are the focus of this report from here forward and will be referred to as having MCI or AD.

### Prevalence estimations

We estimated yearly prevalence rates of MCI and AD (i.e., for each FY from 2011–2018) among Veterans who were 65 years or older by the end of the FY by applying the case definitions to identify the number of likely and suspected MCI and AD cases as the numerator, divided by the total population who used the VAHS excluding those who had previously died or left the VAHS by the FY. Prevalence rates were presented as percentages. Yearly prevalence rates were also standardized for age and sex to the 2020 US Census [10].

We considered that yearly estimations might omit Veterans who did not consistently visit VA clinics for AD care; however, AD is pathophysiologically an absorbing health state and cannot be cured (i.e., once an individual has AD, they will continue to have AD until death) and, when attributable to AD, MCI can also be considered an absorbing state. Therefore, in a sensitivity analysis, we estimated 4-year period prevalence of AD and MCI, accumulating Veterans meeting case definitions from the prior 3 years through each current FY. By using a uniform 4-year accumulation window, we attempted to avoid creating a numeric artifact whereby the opportunity to accumulate cases from preceding years would have been greater for the later vs the earlier FYs within the study window, potentially leading to an exaggeration of the increasing prevalence trend. The 4-year period prevalence was reported for FYs 2014–2018 by accumulating prevalent cases of MCI or AD over 4 year periods.

### Cognitive test score-based severity distribution

Among Veterans fulfilling the AD case definition according to the cumulative 4-year counts described above, available cognitive test scores from the Mini-Mental State Examination (MMSE) and Montreal Cognitive Assessment (MoCA), the most frequently used assessments for dementia in the VAHS, were extracted from notes over the entire study period. Veterans with scores were classified with mild, moderate, or severe AD based on published ranges/cut-offs: 21–24 for MMSE and 18–25 for MoCA corresponded to mild; 13–20 for MMSE and 11–17 for MoCA corresponded to moderate;≤12 for MMSE and≤10 for MoCA corresponded to severe [11–13]. Scores of≥25 and≥26 for MMSE and MoCA, respectively, were classified as “non-dementia”. For each FY, the Veteran’s last score was reported if multiple cognitive tests were administered in that FY. In order to determine AD severity stage distribution by FY, we divided the number of Veterans classified with a given AD stage in that FY by all Veterans who met the AD case definition and had scores that could be extracted from their notes in that FY.

### Statistical methods

The yearly and cumulative prevalence estimates are presented for all Veterans with MCI or AD. Yearly prevalence estimates were stratified by demographic subgroups: age (65–69, 70–74, 75–79, 80–84, 85–89, 90–94, and 95+ years), sex (female, male), age by sex, race (Asian, Pacific Islander, Black, Native American/Alaskan, White), and ethnicity (Hispanic, non-Hispanic).

## RESULTS

### Veterans with MCI and AD

**Fig. 1:**
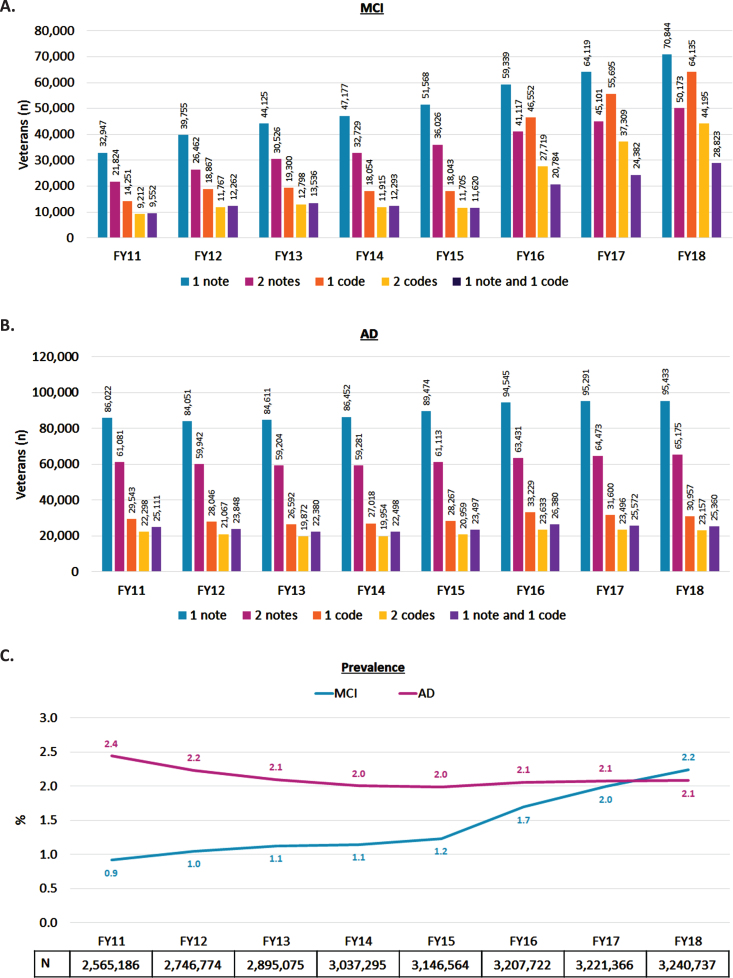
Numbers of Veterans defined by clinical notation or ICD coding recorded with (A) MCI and (B) AD. Yearly (FY 2011–2018) prevalence^a^ of MCI and AD in the VAHS (C). ^a^Proportion of Veterans meeting the MCI or AD case definitions (i.e., 2 qualifying clinical notes, 2 qualifying diagnostic codes, or 1 qualifying note plus 1 qualifying code) in each FY. The N-value listed under each FY represents all Veterans aged 65 years or older who received outpatient or inpatient care in the VAHS in that FY, excluding those with date of death.

Over the 8-year study (FY 2011–2018), 5,229,249 Veterans aged≥65 years received care in the VAHS. Overall, 274,736 and 469,569 Veterans were identified based on a rule-based algorithm as having at least 1 clinical note for MCI and AD, respectively; 201,211 and 149,779 Veterans had at least 1 diagnostic code for MCI and AD, respectively. A total of 147,106 and 207,225 met case definitions for likely MCI and AD, respectively. When suspected cases with only 1 qualifying note/code were combined with likely cases, a total of 283,816 and 400,148 Veterans with MCI and AD were respectively identified, indicating nearly doubled case numbers if all singular MCI- or AD-specific encounters were counted. The distribution of Veterans identified by notes and/or codes by FY is shown in Fig. 1A and 1B. The number of Veterans with MCI recorded by either notes or codes rose from 2011–2018, with more marked increases in the use of codes; the number of Veterans with recorded AD was generally stable from 2011–2018. In each FY, the number of Veterans with MCI based on 2 notes≥30 days apart was consistently higher than the number based on 2 codes≥30 days apart, although the gap was less pronounced from 2016 onward. The number of Veterans with AD in each FY based on 2 notes (ranging from 59,204–65,175 Veterans) was nearly triple the number based on 2 codes (ranging from 19,872–23,633 Veterans). In FY 2018, Veterans meeting the case definitions for MCI (*n* = 85,629) and AD (*n* = 81,543) had a mean (SD) age of 77 (8) and 80 (9) years, respectively (Supplementary Table 1). In both MCI and AD groups, Veterans were predominantly male (97%), White (77–78%), and Non-Hispanic (89–90%).

### Yearly prevalence of MCI and AD

**Fig. 2:**
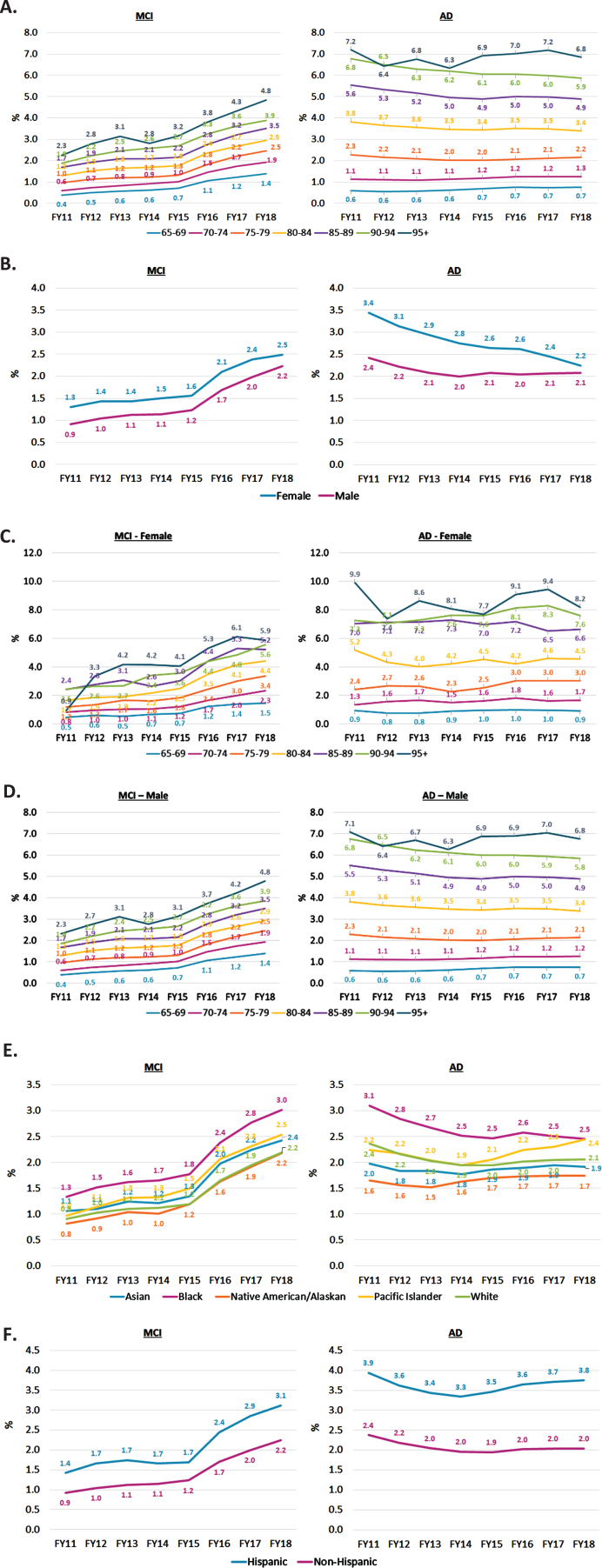
Yearly prevalence^a^ of MCI and AD in the VAHS (FY 2011–2018) stratified by (A) Age group (years); (B) Sex; (C) Age group (years) by female sex; (D) Age group (years) by male sex; (E) Race; (F) Ethnicity. ^a^Proportion of Veterans in each subgroup meeting the MCI or AD case definitions (i.e., 2 qualifying clinical notes, 2 qualifying diagnostic codes, or 1 qualifying note plus 1 qualifying code) in each FY.

From FY 2011 to 2018, the yearly prevalence of MCI in Veterans more than doubled from 0.9% to 2.2%, while yearly prevalence of AD decreased slightly from 2.4% to 2.1% (Fig. 1C). Yearly MCI and AD prevalence rates were consistently higher in older than younger age groups (Fig. 2A). In FY 2018, the yearly prevalence of MCI ranged from 1.4% in Veterans aged 65–69 years to 4.8% in those≥95 years; yearly prevalence of AD ranged from 0.7% in Veterans aged 65–69 years to 6.8% in those≥95 years. Female Veterans had a slightly higher prevalence of MCI and AD than males each year (Fig. 2B). For each age group, female sex was associated with higher prevalence of both MCI and AD, with more pronounced sex differences in older age groups (Fig. 2C, D). The yearly prevalence rates of MCI and AD were generally highest in Black Veterans than other races (Fig. 2E). In FY 2018, yearly MCI prevalence in Black, Pacific Islander, Asian, White, and Native American/Alaskan Veterans was 3.0%, 2.5%, 2.4%, 2.2%, and 2.2%, respectively; yearly AD prevalence across these groups was 2.5%, 2.4%, 2.1%, 1.9%, and 1.7%, respectively. Hispanic Veterans had a higher yearly prevalence of MCI and AD than non-Hispanics from 2011–2018, with a wider gap in AD (Fig. 2F). In FY 2018, for Hispanic vs non-Hispanic Veterans, MCI prevalence was 3.1% vs 2.2%, respectively; AD prevalence was 3.8% vs 2.0%, respectively. Yearly prevalence standardized to the 2020 US population is included in Supplementary Figure 1.

### 4-year period prevalence of MCI and AD

**Fig. 3:**
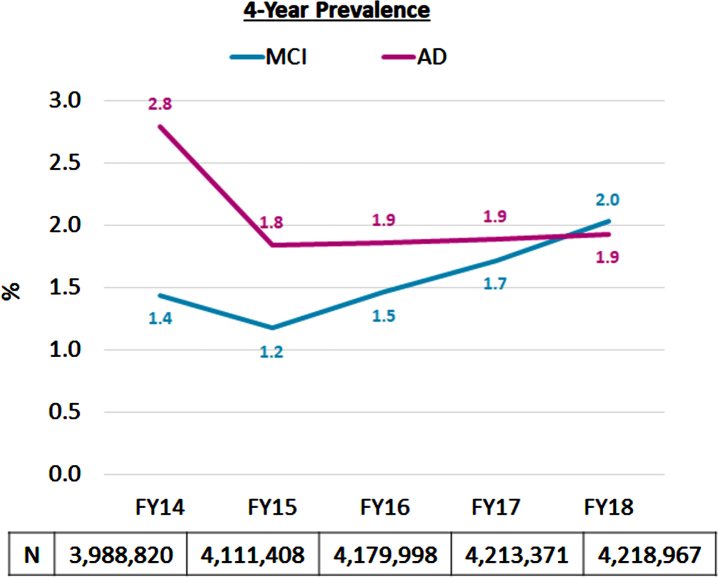
Four-year period prevalence^a^ of MCI and AD in the VAHS (FY 2014–2018). ^a^Proportion of Veterans meeting the MCI or AD case definitions (i.e., 2 qualifying clinical notes, 2 qualifying diagnostic codes, or 1 qualifying note plus 1 qualifying code) in each FY plus those meeting the case definitions in the prior 3 FYs. The N-value listed under each FY represents all Veterans aged 65 years or older who received outpatient or inpatient care in the VAHS in that FY, excluding those with date of death.

The 4-year period prevalence of MCI in Veterans decreased from 1.4% in FY 2014 to 1.2% in FY 2015, followed by a steady increase to 2.0% in FY 2018 (Fig. 3). The 4-year period prevalence of AD decreased from 2.8% in FY 2014 to 1.8% in FY 2015, and then was stable at 1.9% from FYs 2016–2018.

### Distribution of cognitive test score-based AD severity

**Fig. 4:**
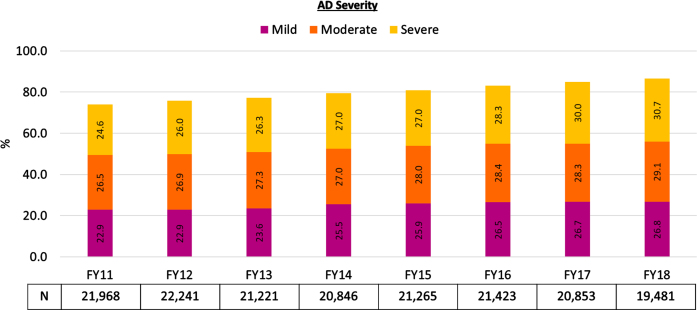
Proportion of Veterans with cognitive score-based AD staging in the mild, moderate, or severe ranges. ^a^MMSE, Mini-Mental State Examination; MoCA, Montreal Cognitive Assessment. ^a^The following proportions of Veterans had cognitive test scores in non-dementia ranges: 26% in FY 2011; 24.2% in FY 2012; 22.8% in FY 2013; 20.5% in FY 2014; 19.1% in FY 2015; 16.8% in FY 2016; 15% in FY 2017; 13.4% in FY 2018. The N-value listed under each FY represents all Veterans in that FY who (1) met the accumulating case definition for an AD diagnosis and (2) had cognitive test scores from MMSE or MoCA that could be extracted from their clinical notes.

Across the FYs 2011–2018 study period, 45% (87,039/193,012) of Veterans meeting the AD case definition based on the 4-year cumulative counts had MMSE and/or MoCA scores in their notes. The demographic characteristics of Veterans with or without cognitive scores were generally comparable (Supplementary Table 1). The distribution of score-based AD stage across the study period is shown in Fig. 4. Among Veterans who met our criteria for AD diagnosis and had MMSE or MoCA cognitive test scores that could be extracted from their clinical notes, between 2011 to 2018, the proportion with mild AD increased from 22.9% to 26.8%, the proportion with moderate AD increased from 26.5% to 29.1%, and the proportion with severe AD range increased from 24.6% to 30.7%. The proportion of such veterans who had scores in the non-dementia range declined from approximately 26% in FY 2011 to 13% in FY 2018.

## DISCUSSION

This retrospective study of the VAHS EHR illustrated the prevalence of Veterans aged 65 years or older with MCI and AD and the trend of prevalence estimates from FYs 2011–2018. We found that yearly prevalence of MCI in US Veterans increased from 0.9% to 2.2%, while yearly prevalence of AD decreased from 2.4% to 2.1%. For both MCI and AD, yearly prevalence rates were higher in female, older, Black, and Hispanic Veterans compared to male, younger, White, and non-Hispanic Veterans. Four-year cumulative prevalence trends from FYs 2014–2018 were generally comparable to yearly trends: cumulative MCI prevalence increased from 1.4% in 2014 to 2.0% in 2018, while cumulative AD prevalence decreased from 2.8% in FY 2014 to approximately 1.9% during FYs 2015–2018. This suggests that Veterans identified with MCI or AD from yearly estimation were generally stable in VAHS clinical practice after excluding those who died or left VAHS healthcare services. Note-based identification consistently yielded more cases than reliance on codes alone. Given that the diagnoses of AD may be underreported and that diagnostic codes for AD may be underutilized [8, 9, 14], we believe that the examination of both notes and codes improves case estimation. Nonetheless, our estimate of 81,543 Veterans with likely AD in FY 2018 was less than half the Department of Veteran’s affairs projection of 167,954 for FY2022 [5]; this could be because the VA report appeared to be based on projections from 2013 populations, whereas our estimates are from the current VAHS EHR utilizing strict definitions requiring 2 qualifying AD-specific codes/notes.

Although direct comparisons cannot be made due to methodologic differences, it is of interest to note that the overall prevalence trend of increasing MCI and decreasing AD in our study is consistent with trends in another recent investigation of the VAHS by Dinesh et al. [6]. However, the previous prevalence estimates were lower than those in our study: in 2018, Dinesh et al reported MCI and AD prevalence rates of approximately 1% and < 0.7%, respectively, whereas our 2018 prevalence estimates for MCI and AD were both approximately 2%. Although both studies used the VAHS database, there are important methodological differences: (1) Dinesh et al. included all Veterans aged 50 years and older, whereas we examined Veterans aged 65 years and older; (2) case identification in the prior study was based solely on having ≥2 codes whereas our case definition required 2 qualifiers, whether they be notes or codes, 30 days apart.

The MCI and AD prevalence rates estimated in our investigation as well as rates reported by Dinesh et al. [6] are markedly lower than 2020 US Census-adjusted rates from the Chicago Health and Aging Project (CHAP) reported by Rajan et al. [15]. The prevalence of MCI (all types) and of clinical AD in CHAP was approximately 23% and 11%, respectively among Americans aged≥65 years. These substantial differences are likely due to vastly different methodologies and populations: CHAP uses active identification with an in-person survey, involving in-home interviews and cognitive tests administered to over 10,000 individuals in the South Side of Chicago, whereas our retrospective investigation uses clinical documentation of MCI/AD in the VAHS EHR, representing a nationwide US Veteran sample. That said, our study may better represent Veterans in the healthcare stream seeking clinical evaluation and treatment for MCI and AD. It is conceivable that a substantial number of Veterans with prevalent MCI or AD are not seeking care and thus their MCI or AD has not been screened/detected. Veterans meeting the case definition for MCI or AD were the focus of the current study; however, we considered that the presence of only 1 note/code could be indicative of evaluation for these conditions (i.e., suspected cases)–a potentially important population for future investigations exploring VAHS clinical practice patterns. The case definition based on notes and/or codes may reflect not only patient clinical profiles but perhaps, more importantly, clinical/institutional practice patterns and patient health seeking behavior. Thus, our prevalence estimates likely provide a realistic estimation of patients who may be examined for eligibility to qualify for newly approved anti-amyloid therapy.

### Limitations

We had assumed that diagnostic codes often reflect clinical workup rather than clinical judgment (i.e., rule-out coding); however, our data suggest relatively sparing use of codes in administrative reporting. Far more cases were identified by notes than by codes. Another consideration is that in the VAHS, codes are used only for administrative/clinical purposes, differing from traditional claims databases where codes are required for reimbursement. We extracted MCI/AD diagnoses from notes to address limitations of using codes alone. However, we found that text extracted from notes did not yield substantial contextual information regarding clinician judgments without a manual review/interpretation of the notes by clinicians. Even so, our iterative processes for qualifying notes with MCI/AD key words had PPVs≥80%. Despite limitations, our case definition separating likely MCI/AD cases from the more sporadically assessed suspected cases appears to be reasonable.

Epidemiological estimates can differ widely based on how MCI is defined [16]. Thus, another limitation is that we did not specifically identify MCI due to AD; there is no diagnostic code for “MCI due to AD” and our notes-based method captured all-cause MCI. Additionally, our AD severity relied on cognitive testing, which does not capture behavioral, psychiatric, or physical dimensions that could impact clinicians’ severity assessments; nonetheless, score-based classification was practical given that cognitive tests are commonly used and scores are readily extracted from notes. The observation that > 10% of our AD cases carry cognitive test scores in the non-dementia range suggests that clinicians consider additional factors when assigning an AD diagnosis. A prior VAHS-based investigation compared cognitive score-based AD severity assessment to clinician’s subjective assessments (both extracted from notes) and reported that almost half of assessments were discordant [13]. Differences between score-based and clinical AD assessments may be due to healthcare system factors such as clinician and clinic types. Finally, findings in this predominantly male, White, non-Hispanic Veteran population may not be generalizable to the current US population at risk for AD; however, prevalence rates standardized to the 2020 US census are provided in the Supplementary Material.

### Conclusions

This is the first attempt to estimate prevalence of MCI and AD in the US Veterans aged 65 years or older based on combining clinical notes and diagnostic codes. From 2011 to 2018, the prevalence of MCI in Veterans increased, while the prevalence of AD decreased. In 2018, there were 85,629 Veterans identified with likely MCI and 81,543 with likely AD in the VAHS; the corresponding yearly prevalence estimates for MCI and AD were 2.2% and 2.1%, respectively. The relative distribution of cognitive test score-based AD severity remained generally stable over time. Accurate prevalence estimation is an important starting point for improving resource allocation for AD care. Our findings suggest there may be underdetection or delayed AD diagnosis in the VAHS. Future research can explore whether seemingly low prevalence estimates may be due to lack of healthcare seeking by patients/caregivers, barriers in referral processes, and/or lack of specialty care access, which could impede patients receiving innovative medicines. The current analysis and follow-up investigations will be important in helping the VAHS identify clinical priorities/gaps in clinical practice and system characteristics that may support or hinder patient access to new AD medicines.

### AUTHOR CONTRIBUTIONS

Byron J. Aguilar (Conceptualization; Data curation; Formal analysis; Investigation; Methodology; Validation; Writing – review & editing); Guneet K. Jasuja (Data curation; Formal analysis; Methodology); Xuyang Li (Data curation; Formal analysis; Methodology); Ekaterina Shishova (Data curation; Formal analysis; Methodology); Natalia Palacios (Data curation; Formal analysis; Methodology); Dan Berlowitz (Conceptualization; Data curation; Formal analysis); Peter Morin (Conceptualization; Formal analysis); Maureen K. O’Connor (Data curation; Formal analysis); Andrew Nguyen (Data curation; Formal analysis); Joel Reisman (Data curation; Formal analysis; Methodology); Yue Leng (Formal analysis; Writing – review & editing); Raymond Zhang (Formal analysis; Methodology); Amir Abbas Tahami Monfared (Conceptualization; Investigation); Quanwu Zhang (Conceptualization; Investigation; Methodology; Resources; Writing – review & editing); Weiming Xia (Conceptualization; Formal analysis; Funding acquisition; Investigation; Project administration; Software; Supervision; Writing – review & editing).

## Supplementary Material

Supplementary Material

## Data Availability

The data supporting the findings of this study are available within the article and/or its supplementary material.
