# Supplementary material for: Prevalence of Mild Cognitive Impairment and Alzheimer’s Disease Identified in Veterans in the United States
Source: J Alzheimers Dis. 2024 May 28;99(3):1065–75. doi: 10.3233/JAD-240027 (PMC11191444; doi:10.3233/JAD-240027)
Supplement: Supplementary Material [file jad-99-jad240027-s001.pdf]

Supplementary Material

Prevalence of Mild Cognitive Impairment and Alzheimer's Disease Identified in Veterans in the United States

Supplementary Table 1. Demographic Characteristics of Veterans with AD with and without Cognitive Tests (FY 2018)

**Demographics (FY 2018)**	**AD with cognitive test (N = 87,039)**	**AD without cognitive test (N = 105,973)**
**Mean±SD Age, y**	83 (8.5)	84 (8.9)
65-69	3,766 (4.3%)	6,320 (6.0%)
70-74	13,420 (15.4%)	14,440 (13.6%)
75-79	11,495 (13.2%)	11,097 (10.5%)
80-84	14,101 (16.2%)	15,380 (14.5%)
85-89	20,336 (23.4%)	24,066 (22.7%)
90-95	15,698 (18.0%)	21,769 (20.5%)
95+	8,223 (9.4%)	12,901 (12.2%)
**Sex, %**		
Male	84,736 (97.4%)	103,348 (97.5%)
Female	2,303 (2.6%)	2,625 (2.5%)
**Race, %**		
White	66,277 (76.1%)	82,820 (78.2%)
Black	11,785 (13.5%)	10,455 (9.9%)
Native Hawaiian or Other Pacific Islander	923 (1.1%)	859 (0.8%)
Alaskan Native or Native American	518 (0.6%)	689 (0.7%)
Asian	600 (0.7%)	430 (0.4%)
Missing/Declined/Unknown	6,936 (8.0%)	10,720 (10.1%)
**Ethnicity, %**		
Non-Hispanic	77,996 (89.6%)	92,212 (87.0%)
Hispanic	5,046 (5.8%)	7,046 (6.6%)
Missing/Declined/Unknown	3,997 (4.6%)	6,715 (6.3%)

Supplementary Figure 1. Yearly Prevalence of MCI and AD Standardized to the US 2020 Population

A. Overall

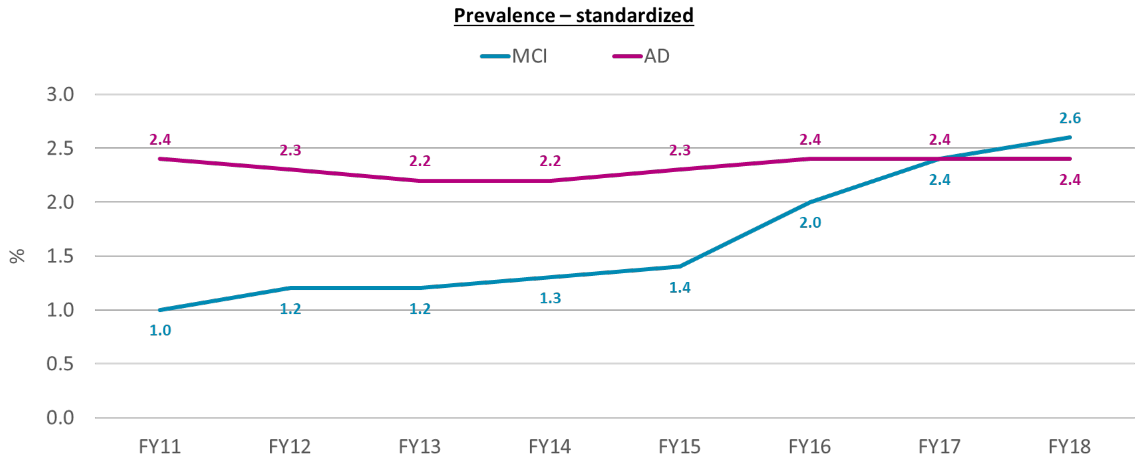

B. Sex

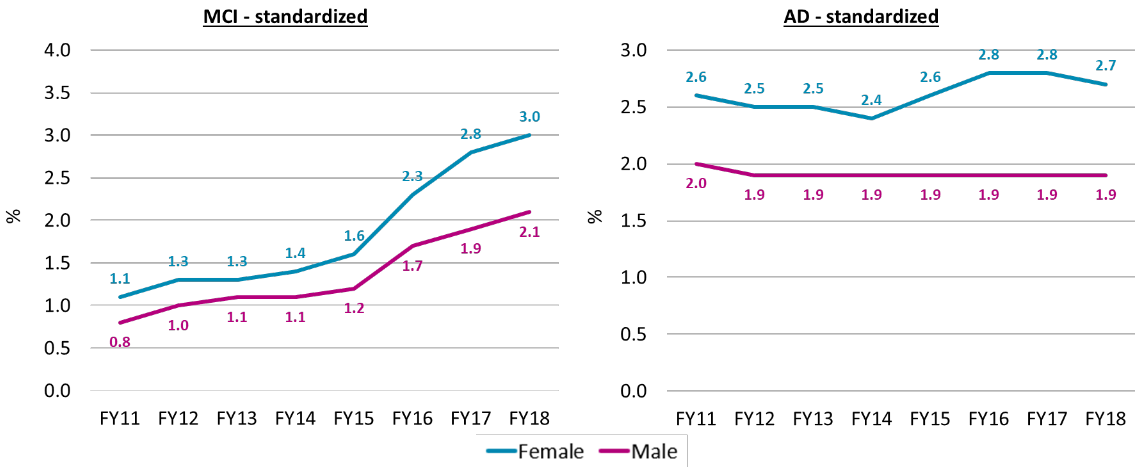

C. Race

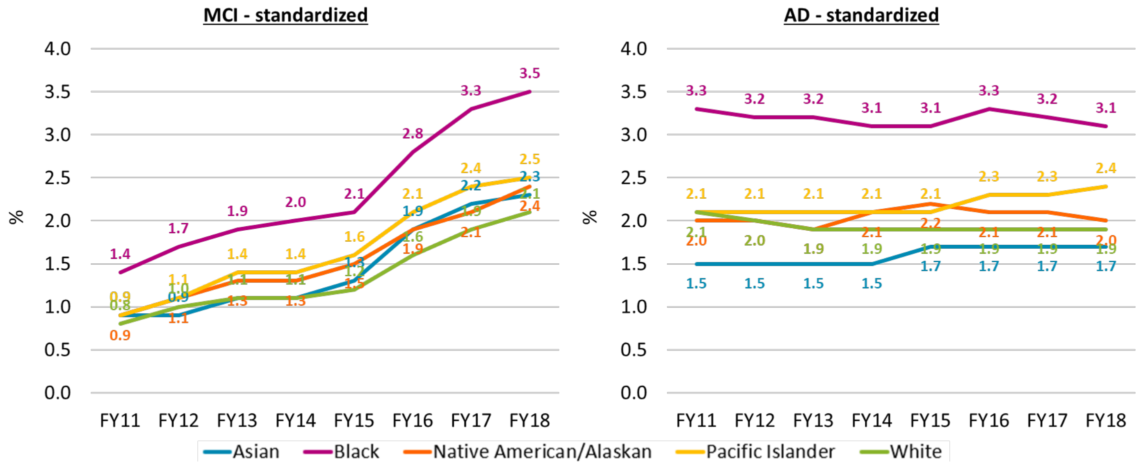

D. Ethnicity

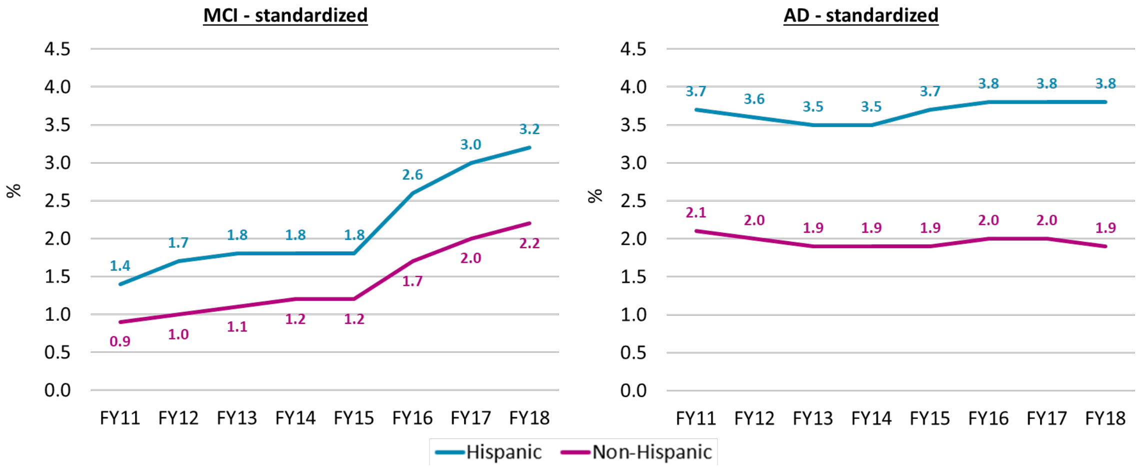

Yearly prevalence rates were further standardized for age and sex to the 2020 US Census-based general population.
